# Not All Words Are Equally Acquired: Transitional Probabilities and Instructions Affect the Electrophysiological Correlates of Statistical Learning

**DOI:** 10.3389/fnhum.2020.577991

**Published:** 2020-09-23

**Authors:** Ana Paula Soares, Francisco-Javier Gutiérrez-Domínguez, Margarida Vasconcelos, Helena M. Oliveira, David Tomé, Luis Jiménez

**Affiliations:** ^1^Human Cognition Lab, CIPsi, School of Psychology, University of Minho, Braga, Portugal; ^2^Psychological Neuroscience Lab, CIPsi, School of Psychology, University of Minho, Braga, Portugal; ^3^Department of Audiology, School of Health, Polytechnic Institute of Porto, Porto, Portugal; ^4^Brain Research Institute (BRI), Porto, Portugal; ^5^Department of Psychology, University of Santiago de Compostela, Santiago de Compostela, Spain

**Keywords:** statistical learning, transitional probabilities, implicit learning, explicit learning, exposure time, electrophysiological correlates, word segmentation, artificial language

## Abstract

Statistical learning (SL), the process of extracting regularities from the environment, is a fundamental skill of our cognitive system to structure the world regularly and predictably. SL has been studied using mainly behavioral tasks under implicit conditions and with triplets presenting the same level of difficulty, i.e., a mean transitional probability (TP) of 1.00. Yet, the neural mechanisms underlying SL under other learning conditions remain largely unknown. Here, we investigated the neurofunctional correlates of SL using triplets (i.e., three-syllable nonsense words) with a mean TP of 1.00 (*easy “words”*) and 0.50 (*hard “words”*) in an SL task performed under incidental (implicit) and intentional (explicit) conditions, to determine whether the same core mechanisms were recruited to assist learning. Event-related potentials (ERPs) were recorded while participants listened firstly to a continuous auditory stream made of the concatenation of four easy and four hard “words” under implicit instructions, and subsequently to another auditory stream made of the concatenation of four easy and four hard “words” drawn from another artificial language under explicit instructions. The stream in each of the SL tasks was presented in two consecutive blocks of ~3.5-min each (~7-min in total) to further examine how ERP components might change over time. Behavioral measures of SL were collected after the familiarization phase of each SL task by asking participants to perform a two-alternative forced-choice (2-AFC) task. Results from the 2-AFC tasks revealed a moderate but reliable level of SL, with no differences between conditions. ERPs were, nevertheless, sensitive to the effect of TPs, showing larger amplitudes of N400 for easy “words,” as well as to the effect of instructions, with a reduced N250 for “words” presented under explicit conditions. Also, significant differences in the N100 were found as a result of the interaction between TPs, instructions, and the amount of exposure to the auditory stream. Taken together, our findings suggest that triplets’ predictability impacts the emergence of “words” representations in the brain both for statistical regularities extracted under incidental and intentional instructions, although the prior knowledge of the “words” seems to favor the recruitment of different SL mechanisms.

## Introduction

The environment in which we live is characterized by a series of sounds, objects, and events that do not occur randomly. The ability to pick up these regularities in time and space is a fundamental skill of our cognitive system to structure the world in a regular and predictable way, and to constantly develop adaptive responses to it (see Reber, 1989, 2013; Thiessen et al., 2013; Erickson and Thiessen, 2015). The mechanism by which we are capable of extracting those regularities, even without intention and/or awareness of doing it, is called statistical learning (SL). This term was coined by Saffran et al. (1996a) in a article showing that 8-month-old infants were capable of computing the probability of a given segment (i.e., a syllable) to be followed by another segment (another syllable) in a continuous stream made up of the concatenation of three-syllable nonsense words generated from an artificial language (e.g., “*tokibu*,” “*gikoba*,” “*gopila*,” “*tipolu*”) repeated in random order with no pauses between each other (e.g., “*gikobatokibutipolugopilatokibu*”), and to use these computations, known as transitional probabilities (TPs), to discover word’s boundaries. Note that in that artificial language, as in natural languages, the TPs between syllables composing a given “word” (e.g., “*tokibu*,” “*gikoba*”) were higher than the TPs of syllables overlapping two “words” (e.g., “*bugiko*”), hence making TPs a reliable cue for words’ segmentation.

Since this seminal study, several other studies using the same task, also known as triplet embedded task, have shown that SL can also be observed in younger infants (e.g., Kirkham et al., 2002; Teinonen et al., 2009; Bulf et al., 2011), older children, and adults (e.g., Saffran et al., 1996b, 1997, 1999; Fiser and Aslin, 2002; Saffran and Wilson, 2003; Turk-Browne et al., 2005; Endress and Mehler, 2009; Arciuli and Simpson, 2012), and not only with syllables as stimuli, but also with tones, geometric shapes, and symbols. Nonetheless, even though these studies provide strong evidence for the view that individuals from different ages are sensitive to the statistical properties embedded in different inputs (see, however, Frost et al., 2015; Siegelman and Frost, 2015 for modality and stimulus specificities in SL), such findings, obtained mainly from standard SL experiments, provide little evidence about both the process of learning and the nature of the representations that arise from the SL tasks (see Batterink and Paller, 2017; Batterink et al., 2019 for recent discussions).

In a typical SL experiment, participants are asked to perform a familiarization phase, followed by a two-alternative forced-choice (2-AFC) task in which participants are asked to choose the most familiar stimulus out of pair composed by a “word” from the artificial language and a foil made up of the same syllables but never presented during the exposition. Above-chance performance indicates that SL had occurred, but it does not inform about the processes by which participants track the statistical regularities embedded in the input as exposition unfolds. A good strategy to assess this learning process could be using online measures as event-related potentials (ERPs) registered during the familiarization phase, as they are highly sensitive to the time course of processing (millisecond precision), and are less affected by other meta-cognitive or strategic factors that might affect SL results (for a discussion see Daltrozzo and Conway, 2014; and also Siegelman et al., 2017a).

Thus, it is not surprising that recent studies have been using online (neural) and not only post-learning offline measures of SL (e.g., 2-AFC) to study the processes and mechanisms that underlie the extraction of the statistical regularities embedded in the input and also to shed light on other controversial issues largely unexplored in the SL literature, as the nature of representations that arise from SL tasks (e.g., Batterink et al., 2015a, 2019; Batterink and Paller, 2017; Batterink et al., 2019; Kóbor et al., 2018, 2019; Batterink, 2020; Horváth et al., 2020; see Batterink et al., 2019; and Daltrozzo and Conway, 2014 for recent reviews). This strongly contrasts with what has been investigated in the related implicit learning field (see Perruchet and Pacton, 2006, and also Christiansen, 2019) where a significant amount of research has been devoted to examining the type of representations and the (implicit vs. explicit) nature of the knowledge emerging from tasks such as the artificial grammar learning (AGL) task (Reber, 1967) or the serial reaction time (SRT) task (Nissen and Bullemer, 1987) or any version of it, either using subjective confidence scales [see for instance Jiménez et al., 2020 or Soares (under review) for recent examples] or dissociating these two types of knowledge through the manipulation of the instructions. Although studies using AGL and SRT tasks have yielded somewhat contradictory results regarding the effects of explicit instructions on learning, with studies showing either detrimental (e.g., Reber, 1976; Howard and Howard, 2001), null (e.g., Dulany et al., 1984; Dienes et al., 1991; Jiménez et al., 1996; Song et al., 2007; Sanchez and Reber, 2013), or beneficial effects (e.g., Howard and Ballas, 1980; Reber et al., 1980), accumulated evidence suggests that explicit instructions might enhance performance, particularly when the stimuli are not presented at a high speed when the to-be-learned regularities are simple, and when specific information about these regularities is provided to the participants before the familiarization phase (e.g., Howard and Ballas, 1980; Reber et al., 1980, see Arciuli et al., 2014 for a discussion). Note that even though in the implicit learning field the terms “incidental” and “intentional” are often used to refer to participants’ passive vs. active orientation toward the encoding and retrieval processes, whereas the labels “implicit” and “explicit” are reserved to the description of the resulting representations (see Shanks, 2005), for simplicity we will stick to the same labels for both uses, using implicit vs. explicit conditions to refer to task-instruction manipulations, even without assuming a one-to-one correspondence between the type of instructions and resulting outcomes (i.e., explicit instructions do not immediately qualify the outcomes as explicit, and vice versa).

In the SL literature, the few studies conducted so far on the type of representations emerging from sequential learning tasks either in the auditory or visuomotor modality suggest that both implicit and explicit representations might arise from SL tasks (e.g., Turk-Browne et al., 2009, 2010; Franco et al., 2011; Bertels et al., 2012, 2015; Batterink et al., 2015a,b; Kóbor et al., 2018, 2019; Horváth et al., 2020). For instance, Batterink et al. (2015a) recorded behavioral (RTs/accuracy) and ERP responses while participants performed two post-learning tasks: a speeded target detection task, aimed to assess SL indirectly as it asks participants to detect as fast and accurately as possible a specific syllable within a continuous speech stream, and the abovementioned 2-AFC task combined with a remember/know procedure to assess SL directly under either implicit or explicit conditions. In the implicit group, participants were instructed to listen to the auditory stimuli, whereas in the explicit group participants were informed they would listen to a nonsense language and their task would be to discover where each word began and ended since they would be tested afterward on their knowledge about that language. Results from the target detection task failed to show any significant difference between the implicit and the explicit groups both at behavioral and brain levels. However, participants from both groups were faster at detecting syllables occurring in the later “word” positions than in the initial “word” position, indicative of the *triplet onset effect* observed in previous behavioral SL studies (e.g., Turk-Browne et al., 2005; Kim et al., 2009; Franco et al., 2015; Siegelman et al., 2018). Note that, because TPs within triplets are higher than TPs across triplets’ boundaries, the final syllables within a triplet become more predictable than the syllables at the onset, hence giving rise to faster reaction times (RTs) to the second and third syllables relative to the first one as SL accrues. Consistently, the neural data from the speeded target detection task showed facilitation attributable to SL in the processing of the syllables in the middle and final positions compared to initial syllables. Results from the 2-AFC task showed that accurate responses were associated with subjective feelings of stronger recollection, although explicit stimulus recognition did not correlate either with RTs or electrophysiological effects. These findings led the authors to conclude that dissociable implicit and explicit forms of knowledge accrued in parallel during SL tasks. Even though the failure to observe differences between implicit and explicit conditions is in line with previous findings pointing to null effects of the instructions on a diversity of implicit learning tasks (e.g., Dulany et al., 1984; Dienes et al., 1991; Jiménez et al., 1996; Song et al., 2007; Sanchez and Reber, 2013), these results may also arise from the fact that the information provided in the explicit condition was too vague to impact SL performance positively. Indeed, in a subsequent study with extensive training of the nonsense words used by Batterink et al. (2015a,b), the authors reported significant behavioral and neural differences in words’ processing as a function of the training condition: participants in the explicit condition were faster at detecting predictable targets and marginally slower to detect less predictable targets, relatively to the participants in the implicit condition. In the same vein, ERP results indicated greater involvement of controlled, effortful, processes when the information was acquired explicitly.

Although providing some insights into the extent to which SL recruits the same core mechanisms under implicit and explicit conditions, these ERP studies left open several important issues. For example, because in these studies behavioral and ERP data were collected after the familiarization phase, they reflect more the outcome of SL than the processes underlying that learning. Previous ERP studies provided a fine-grained measure of how SL occurs in the brain. Sanders et al. (2002; see also Sanders et al., 2009) reported some of the first studies that collected ERPs while participants were familiarized with a continuous stream made of three-syllable nonsense words. They found that initial syllables elicited larger N100 and N400 potentials than syllables in latter positions, which was interpreted as an index of the triplet onset effect in the brain. Subsequent studies (e.g., Cunillera et al., 2006, 2009; De Diego Balaguer et al., 2007; Abla et al., 2008; Abla and Okanoya, 2009; Teinonen et al., 2009; François et al., 2014; Mandikal-Vasuki et al., 2017a) found similar results, which provide further evidence to interpret these ERP components, particularly the N400, as reflecting the neural signature of “words” segmentation in the brain. Other ERP components have also been reported in response to SL tasks during the familiarization phase. For instance, François et al. (2017) and also Mandikal-Vasuki et al. (2017b) recently reported a negative ERP component peaking at ~250 ms (N250) as indexing the recruitment of greater attentional resources from triplet onsets. Kóbor et al. (2018) have also reported an effect on N2, correlated both to statistical and sequence learning in an SRT-like task. The effect consists of an attenuated N2 amplitude in pattern (vs. random) presentations, which has been also related to the deployment of more attentional resources. In the same latency range, Koelsch et al. (2016) and Tsogli et al. (2019) recently reported an analog of the mismatch negativity (MMN) wave, called the statistical MMN (sMMN), to index the automatic change detection processes based on implicit extraction of statistical regularities embedded in the input.

Furthermore, it is important to note that the vast majority of previous ERP studies did not provide information regarding the changes that the neural correlates of SL might undergo as exposition to the stream unfolds (for exceptions see Abla et al., 2008; François et al., 2014; Batterink and Paller, 2017). This is particularly important because recent studies suggest that the learning of the statistical regularities embedded in the input occurs during the first trials/few minutes of familiarization (see De Diego Balaguer et al., 2007; Turk-Browne et al., 2009). Thus, if the time of exposure is not considered, potential neurofunctional differences between implicit and explicit learning conditions might become undetectable. Indeed, in one of the few ERP studies examining how these components change as exposition to the auditory stream unfolds, Abla et al. (2008) reported that participants who showed the best SL results in the 2-AFC task (i.e., *M* = 90.24% of correct responses; min: 81.37%) revealed an earlier and larger triplet onset effect in the N100 and N400 ERP components, as compared to participants with moderate SL results (i.e., *M* = 72.5% of correct responses; range: 67.38–81.37%) who showed this effect only in the last block of the task, whereas low learners (i.e., *M* = 58.62% of correct responses; max: <67.38%) failed to show any effect.

Finally, following the seminal work of Saffran et al. (1996a), most SL studies have tested SL not only under incidental (implicit) conditions but also using triplets with the same level of difficulty (i.e., TPs = 1.00). Note that a triplet with a TP of 1.00 means that a given syllable only occurs in a word and in a fixed position, thus removing all signs of uncertainty from the input. However, in natural languages, syllables do not follow each other with 100% certainty. For instance, the syllable “cur,” can appear in different words in different positions as in *cur*.va.ture, in.*cur*.sion or re.oc.*cur*, and this change in distributional proprieties can affect SL in important ways (see Thiessen et al., 2013 and also Hasson, 2017 for reviews). Notably, few SL studies have manipulated the TPs of the triplets to test how the brain processes different types of statistical structures (see however Siegelman et al., 2017a). Although lowing TPs will probably make triplets harder to learn, studying how SL occurs under more uncertain conditions, which mimic more closely what occurs in natural environments, will contribute to deepening our understanding of how SL works in a wide range of conditions. Testing how the type of instructions (implicit vs. explicit) provided to the participants modulates the recruitment of different neural processes will also contribute to that aim. Some previous studies by Batterink et al.’s (e.g., Batterink et al., 2015a,b) have tested this effect, but they did it in a between-subject design. However, as Siegelman et al. (2017a; see also Siegelman et al., 2017b) recently pointed out, there is a lot of variability in how different individuals respond to SL tasks, and this makes it advisable to analyze the impact of instructions in a within-participants design.

The current work aimed to directly address these issues by examining the neural (ERP) responses elicited during the familiarization phase of an auditory SL task modeled from Saffran et al. (1996a). The task was performed by the same participants under both incidental (implicit) and intentional (explicit) instructions to determine whether the same core mechanisms are recruited to extract the statistical regularities embedded in the continuous auditory stream as the exposition unfolds. If SL under implicit and explicit conditions elicited the same neural responses, this would provide further evidence to the view that they probably rely on the same core mechanisms. In contrast, if a different pattern emerges in each condition, this would support the view that explicit and implicit representations might be mediated by different mechanisms operating in parallel to allow more effective processing. It is also possible that, even though the same basic pattern of results will emerge, differences in the learning dynamics across time will still be observed, with effects arising earlier under explicit than under implicit instructions. Additionally, words’ predictability was also manipulated by using three-syllable nonsense words with a TP of 1.00 (*easy “words”*) or 0.50 (*hard “words”*) presented randomly in two blocks of ~3.5-min each (i.e., 30 repetitions of each “word”) to examine how ERP components might change over time. This exposure time (i.e., ~7-min in each of the SL tasks) was chosen because previous studies (e.g., De Diego Balaguer et al., 2007; Turk-Browne et al., 2009), suggested that the learning of the statistical regularities embedded in the input occurs during the first trials/few minutes of familiarization as mentioned, and also because we have used a within-subject design meaning that each participant performed both SL tasks, which made the procedure necessarily longer. Behavioral evidence of SL was obtained through a 2-AFC task presented after the familiarization phase of each of the SL tasks (implicit and explicit) as it is one of the most frequent measures of SL adopted in the studies conducted so far (e.g., Saffran et al., 1996b, 1997, 1999; Saffran and Wilson, 2003; Turk-Browne et al., 2009; Arciuli and Simpson, 2012; Batterink et al., 2015b; Batterink and Paller, 2017). Following the reviewed literature, we hypothesized that participants would respond more accurately under explicit than implicit conditions. Differences in behavioral performance under implicit vs. explicit conditions were expected to be greater for the hard than for the easy “words.” Regarding neural responses, we also expected easy and hard “words” to elicit different ERP modulations. Based on the assumption that N100 and N400 enhancements index “word” segmentation, easy “words” were expected to elicit larger amplitudes than hard “words.” Specifically, this amplitude enhancement would be expected to occur selectively in the implicit task, since the higher predictability of the easy “words” would allow the brain to build up representations of these triplets more rapidly than they would do for the hard “words.” This difference could be expected to vanish as the exposure time unfolds. In contrast, in the explicit condition, as participants can take advantage of prior knowledge about the structure and content of the stimuli, ERPs could be similarly elicited to both hard and easy “words,” and the same would be expected to occur at the behavioral level.

## Materials and Methods

### Participants

Thirty-two undergraduate students (25 women, *M*_age_ = 23.4, *SD*_age_ = 5.66) from the University of Minho were recruited for the experiment in exchange for academic credits. All participants were native speakers of European Portuguese, with normal hearing, normal or corrected-to-normal vision, and with no history of disabilities and/or neurological problems. Twenty-nine of the participants were right-handed and three left-handed as assessed by the Edinburgh Handedness Inventory (Oldfield, 1971). Written informed consent was obtained from all participants. The study was approved by the local Ethics Committee (University of Minho, Braga, Portugal, SECSH 028/2018).

### Stimuli

For this experiment, two syllabaries (syllabary A and syllabary B) with 16 unique auditory CV syllables each (e.g., “tu,” “ci,” “da,” “mi,” “ge,” “do’ from syllabary A; and “ga,” “pa,” “be,” “me,” “gu,” “pi” from syllabary B) were created to generate the nonsense words to be used in the implicit and explicit versions of the SL task. Note that, because we used a within-subject design, using syllables coming from two different artificial languages (i.e., without any syllable overlap, although vowels were necessarily repeated across syllabaries) was mandatory to minimize interference effects of the first language over the second language, as observed in other studies (e.g., Gebhart et al., 2009; Franco et al., 2011; Shaqiri et al., 2018). Syllables were produced and recorded by a native speaker of European Portuguese with a duration of 300 ms each. Syllables in each syllabary were organized into eight 3-syllable nonsense words: four easy “words” (*M*_TPs_ = 1.0; *SD* = 0.03), and four hard “words” (*M*_TPs_ = 0.50; *SD* = 0.03) following Siegelman et al.’s (2017b) procedure. For instance, the nonsense word “*tucida*” from syllabary A and the nonsense word “*todidu*” from syllabary B correspond to easy “words” as the syllables they entail only appear in those “words” in the same syllable positions, while the nonsense word “*dotige*” from syllabary A and the nonsense word “*pitegu*” from syllabary B correspond to hard “words’ as the syllables they entail appear in three different words in each of the three-syllable positions (“*tidomi*,” “*migedo*,” “*gemiti*” and “*tepime*,” “*megupi*,” “*gumete*,” respectively). See Table 1 for other examples.

**Table 1:** Three-syllable nonsense words and three-syllable nonsense foils from Syllabary A and Syllabary B.

		Syllabary	
		A	B
Nonsense words	easy	*tucida*	*todidu*
		*bupepo*	*cegita*
		*modego*	*gapabe*
		*bibaca*	*bomaco*
	hard	*dotige*	*pitegu*
		*tidomi*	*tepime*
		*migedo*	*megupi*
		*gemiti*	*gumete*
Nonsense foils	easy	*tumica*	*tomeco*
		*bugego*	*cegube*
		*modopo*	*gapita*
		*bitida*	*botedu*
	hard	*dobage*	*pimagu*
		*tidemi*	*tepame*
		*mipedo*	*megipi*
		*geciti*	*gudite*

The nonsense words were concatenated in a continuous stream with the Audacity^®^ software (1999–2019) with no pauses between syllables (900 ms per nonsense word). Each nonsense word was repeated 60 times in two different blocks of 30 repetitions each (Block 1 and Block 2). In each block, the nonsense words were presented binaurally in random order with the restriction that the same nonsense word or the same syllable will never appear consecutively. The TPs across “word” boundaries were therefore of 0.14. The speech stream was edited to include a randomly superimposed chirp sound (a 0.1 s sawtooth wave sound from 450 to 1,450 Hz) to provide participants with a cover task (i.e., a chirp detection task) to ensure adequate attention to the stimuli as in previous SL studies (e.g., Turk-Browne et al., 2009; Arciuli and Simpson, 2012; François et al., 2014; Bertels et al., 2015; Mandikal-Vasuki et al., 2017a, b). The target sound was programmed to appear in the stream intervals between 2 and 10 s, to prevent it from being used as a word segmentation cue. Depending on the variability of the interval, the stream could contain a total of 43 or 44 chirp sounds to detect during the familiarization phase.

For the test phase (2-AFC task), eight three-syllable foils were also created for each syllabary (see Table 1). The foils were made up of the same syllables used in the easy and hard “words” in each syllabary, although they were never presented together in the stream presented during exposure (*M*_TPs_ = 0.00). Syllables in the foils were presented with the same frequency and syllable positions (initial, medial, and final) as the syllables in the easy and hard “words” to avoid frequency and position confounds. Four lists of materials were created in each syllabary to counterbalance syllables across positions in each type of nonsense words (easy and hard). Participants were randomly assigned to the lists. For convenience, Table 1 presents only the stimuli used in List 1 from Syllabary A and List 1 from Syllabary B.

### Procedure

Participants were first presented with the implicit version of the auditory SL task and, subsequently, with the explicit version, each of them comprising a familiarization phase and a test phase. In the implicit task, participants were instructed to pay attention to the sounds (presented at 60 dB SPL *via* headphones binaurally) because occasionally a “click” sound would appear and they had to detect it as soon and accurately as possible by pressing a button in the keyboard. Following familiarization, participants were informed that the sequences of syllables they had just listened corresponded to a foreign language and were asked to complete a 2-AFC task, i.e., to choose which of the two-syllable sequences (the first or the second) resembled most what they have just heard, by pressing respectively the “z” or the “m” buttons in the keyboard. In half of the trials, the correct “word” was presented firstly while in the other half it was presented second. Participants were informed about the test phase only after completion of the familiarization phase to ensure that learning was implicit. The test phase comprised 64 trials in which each of the eight trained “words” were paired with each of the eight foils from the same syllabary. The 64 trials in the 2-AFC were presented in a random order for each participant. Each trial began with the presentation of a fixation cross for 1,000 ms, after which the first stimulus (“word”/foil) was presented. A 500 ms inter-stimulus interval separated the presentation of both sounds. The next trial begins as soon as participants made a response or 10 s had elapsed.

**Figure 1:**
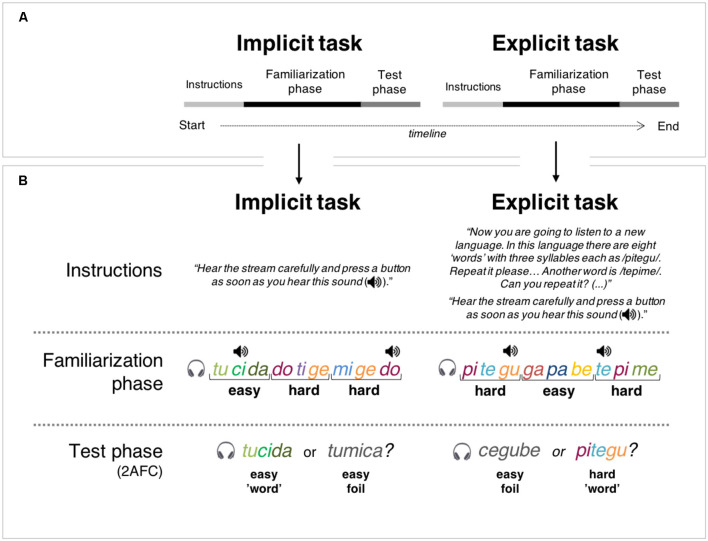
Visual summary of experimental design. Note: Box **(A)** illustrates the timeline of the experimental procedure in which one implicit and, subsequently, one explicit auditory statistical learning (SL) tasks were administered. Each task of the two tasks comprised of three parts: instructions, familiarization phase, and test phase. As can be observed in Box **(B)**, each task was initiated with specific instructions that determined the conditions under which SL occurred: implicit instructions (i.e., without knowledge of the stimuli or the structure of the stream—Implicit task) or explicit instructions (i.e., with explicit knowledge or pre-training on the “words” presented in the stream—Explicit task). In the familiarization phase of both tasks, participants were presented with a continuous auditory stream of four easy and four hard “words,” with chirp sounds (depicted as a speaker icon on the Figure) superimposed over specific syllables. The chirp sounds could emerge at any of three-syllable positions of the “words,” which precluded its use as a cue for stream segmentation. During this phase, participants had to perform a chirp detection cover task. Then, a test phase consisting of a two-alternative forced-choice (2-AFC) task asked participants to indicate which of two syllable-sequences (a “word” and a foil) sounded more familiar considering the stream heard on the familiarization phase.

After a short rest interval, which in no case exceeded 5 min, participants underwent the explicit version of the SL task. This task mimicked the procedure adopted in the implicit SL task except that, before listening to the auditory stream, participants were informed about each of the eight “words” from another foreign language (i.e., the four easy and four hard “words” drawn from the syllabary not used in the implicit version of the task). Specifically, in this phase, each of the eight “words” was presented individually and participants were asked to repeat it correctly before another “word” was presented. At the end of the training phase, participants were asked to pay attention to the auditory stream and to perform a click detection task as in the implicit SL task. Following familiarization, participants performed a 2-AFC task, similar to the one used in the implicit version. The procedure took about 90 min to be completed per participant. Figure 1 depicts a visual summary of the experimental design.

### EEG Data Acquisition and Processing

Data collection was performed in an electric shielded, sound-attenuated room. Participants were seated in a comfortable chair, one meter away from a computer screen. During the familiarization phase, EEG data was also recorded with 64 channels BioSemi Active-Two system (BioSemi, Amsterdam, The Netherlands) according to the international 10–20 system and digitized at a sampling rate of 512 Hz. Electrode impedances were kept below 20 kΩ. EEG was re-referenced off-line to the algebraic average of mastoids. Data were filtered with a bandpass filter of 0.1–30 Hz (zero phase shift Butterworth). ERP epochs were time-locked to the nonsense words” onset, from −300 to 1,000 ms (baseline correction from −300 to 0 ms). Independent component analyses (ICA) were performed to remove stereotyped noise (mainly ocular movements and blinks) by subtracting the corresponding components. After that, epochs containing artifacts (i.e., with amplitudes exceeding ±100 μV) were excluded. EEG data processing was conducted with Brain Vision Analyzer, version 2.1.1. (Brain Products, Munich, Germany).

### Data Analysis

Behavioral and ERP data analyses were performed using IBM-SPSS software (Version 21.0. Armonk, NY, USA: IBM Corporation). For behavioral data, the percentage (%) of correct responses was computed for each of the 2-AFC tasks and separately for the easy and hard “words.” One-sample *t*-tests against the chance level were conducted to determine whether performance in each SL task (implicit vs. explicit) and type of “word” (easy vs. hard) was significantly different from chance. Repeated-measures analysis of variance (ANOVA) was then conducted considering a 2 (instructions: implicit vs. explicit) × 2 (the type of “word”: easy vs. hard) within-subject factors design, to analyze if 2-AFC performance was significantly different across conditions. One participant was excluded from the behavioral analyses due to problems in data recording.

Individual ERPs were averaged separately per condition. Grand averages waveforms were then calculated across individuals in each SL task (implicit vs. explicit), type of “word” (easy vs. hard), and exposure time (Block 1 vs. Block 2). Four participants were excluded from the EEG analyses, one because performed below the chance level in both 2-AFC tasks and three due to artifact rejection (rejected more than 70% of trials). Based on previous literature, mean amplitudes were measured for the following time windows: 80–120 ms (N100), 230–270 ms (N250), and 350–450 ms (N400). To account for the topographical distribution of the abovementioned EEG deflections, mean amplitudes” values were obtained for the topographical regions where amplitudes were maximum, namely: fronto-central region of interest (ROI; F1, Fz, F2, FC1, FCz, FC2, C1, Cz, and C2) for N100 and N250, and central ROI (FC1, FCz, FC2, C1, Cz, C2, CP1, CPz, and CP2) for N400.

Both for behavioral and ERP data, only main or interaction effects that reach statistical or marginal significance levels in comparisons of interest are reported. The Greenhouse–Geisser correction for nonsphericity was used when appropriate. *Post hoc* tests for multiple comparisons were adjusted with Bonferroni correction. Measures of effect size (Eta squared, $\eta_{\text{p}}^{2}$) and observed power (*pw*) for a single effect are reported in combination with the main effects of the condition.

## Results

### Behavioral Data

**Figure 2:**
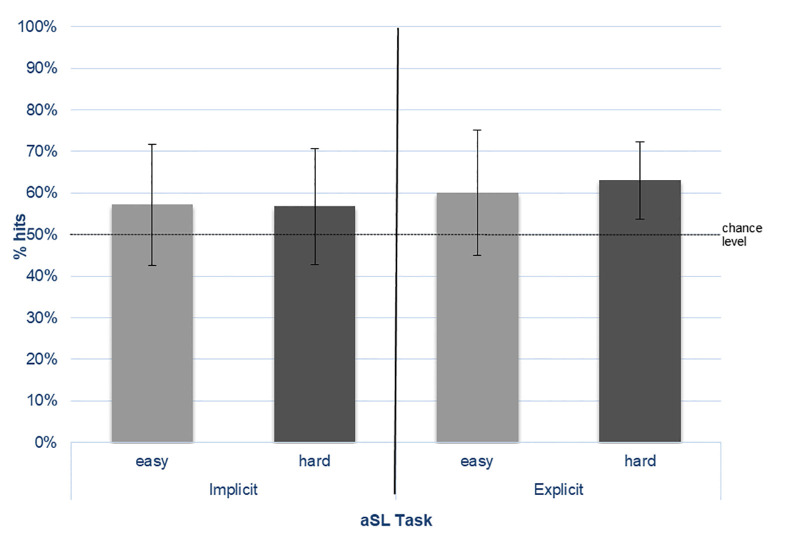
Percentage of correct choices (% hits) for the easy- and hard-nonsense words in the 2-AFC tasks performed under implicit and explicit conditions.

The mean percentage of correct responses (% hits) in the 2-AFC task for the easy and hard “words” per learning condition (implicit vs. explicit) is presented in Figure 2.

The results from the one-sample *t*-tests against chance level showed that, in the implicit task, performance for the easy “words” was 57.1% (*SD* = 14.66) and for the hard “words” was 56.8% (*SD* = 13.94), both differing significantly from chance (easy: *t*_(30)_ = 2.714, *p* = 0.011; hard: *t*_(30)_ = 2.718, *p* = 0.011). In the explicit task, performance for the easy “words” was 60.1% (*SD* = 15.15) and for the hard “words” was 63.0% (*SD* = 9.23), both also above-chance levels (easy: *t*_(30)_ = 3.704, *p* = 0.001; hard: *t*_(30)_ = 7.826, *p* < 0.001). Although the performance was numerically higher in the explicit than in the implicit SL task for both types of “words,” as expected, the results from the repeated measures ANOVA showed that no main or interaction effects reached statistical significance.

### ERP Data

#### N100

**Figure 3:**
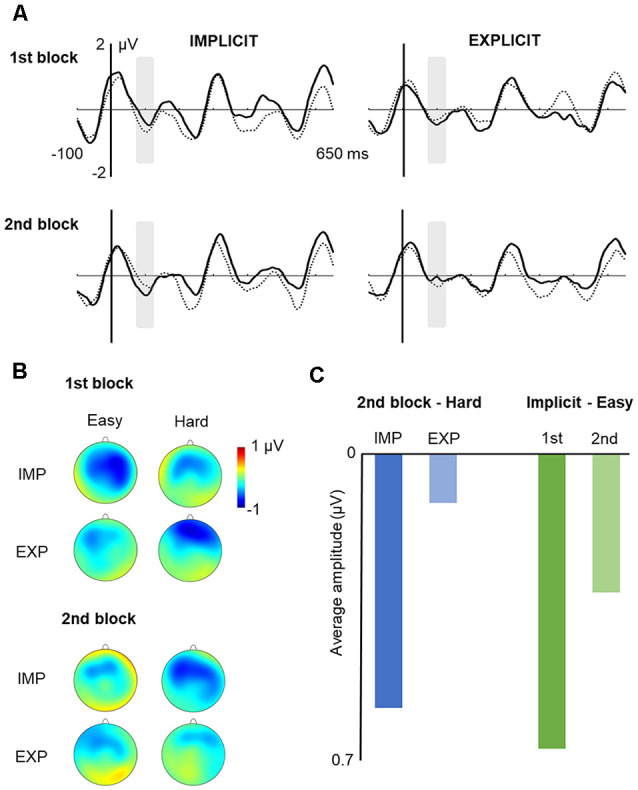
Learning effects on N100 Peak. Note: **(A)** grand average ERPs at the fronto-central ROI (solid line: hard; dotted line: easy). Gray shaded boxes over the event-related potentials (ERPs) indicate the analyzed time window (80–120 ms). **(B)** Voltage maps of each condition: fronto-central distribution of the N100 peak. **(C)** Graphical depiction of the averaged amplitudes for the pairwise comparisons of the triple interaction between SL task, type of “word,” and exposure time.

The ANOVA for the N100 failed to reveal any significant main effect. Although a significant three-way interaction was found, *F*_(1,27)_ = 4.825, *p* = 0.037, $\eta_{\text{p}}^{2}$ = 0.152, the pairwise comparisons revealed to be nonsignificant. However, we found a tendency for hard “words” to exhibit a larger N100 amplitude in the implicit vs. explicit condition in the second block (*p* = 0.059) and a tendency for the easy “words” to exhibit a reduced N100 amplitude in the second vs. the first block in the implicit condition (*p* = 0.058). Further Bayesian analyses (JASP Team, 2020), conducted to test whether these results were more consistent with the existence or with the absence of an effect, provided moderate evidence in favor of its existence both for the first (B_10_ = 1.08) and the second (B_(10)_ = 1.09) pairwise comparisons. Figure 3 depicts the grand-averaged ERPs for the easy and hard “words,” in each learning condition, in Block 1 and Block 2, separately.

#### N250

**Figure 4:**
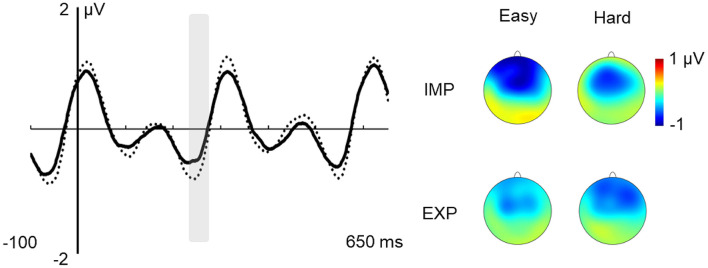
Effect of instructions on the N250 response. Note: grand average ERPs at the fronto-central region of interest (ROI; solid line: explicit; dotted line: implicit). Gray shaded box over the ERPs indicates the analyzed time window (230–270 ms). Voltage maps of each condition: fronto-central distribution of the N250 peak.

In the N250 latency window, there was a main effect of learning condition, *F*_(1,27)_ = 4.775; *p* = 0.038; $\eta_{\text{p}}^{2}$ = 0.150. This effect showed that the N250 amplitude was reduced in the explicit relative to the implicit condition, as can be observed in Figure 4.

#### N400

**Figure 5:**
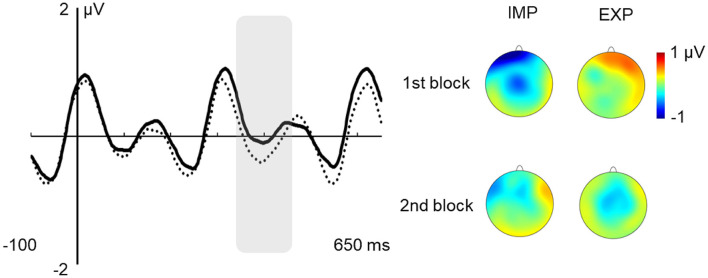
Effect of type of “word” in the N400 Time Window. Note: effect of type of “word” in the N400 time window at the central ROI (solid line: hard; dotted line: easy) and voltage maps of the difference between easy and hard “words.”

The only significant effect in this time window was that of the type of “word,” *F*_(1,27)_ = 4.260; *p* = 0.049; $\eta_{\text{p}}^{2}$ = 0.136, showing greater N400 amplitude for easy vs. hard “words” (see Figure 5).

## Discussion

The present study aimed to examine how SL occurs under conditions of high vs. low words’ predictability and to test whether the same core mechanisms are recruited to extract word-like units from continuous auditory streams under implicit vs. explicit learning conditions. ERP data were recorded while participants performed either an auditory triplet embedded SL task in which statistical regularities had to be abstracted through passive exposure to a continuous “word” stream (implicit condition) or a similar task in which the “words” were explicitly taught before exposure (explicit condition). The TPs between the “words” syllables were respectively of 1.00 or 0.50, thus creating “words” that were easily or hardly predictable in the context of a continuous stream. The “word” stream was presented in two separate blocks of ~3.5-min each to further examine how ERP components might change over time. Behavioral data were collected using a 2-AFC task after the familiarization phase of each SL task. Using a within-subject design aiming to obtain results less affected by individual differences, the current study contributed to shed new light on the processes underlying SL under different learning conditions, involving implicit or explicit instructions, and two degrees of uncertainty (i.e., easy vs. hard “words”).

The results obtained were clear-cut and can be summarized as follows: (i) participants showed a moderate but reliable level of SL, although behavioral performance was neither modulated by words’ predictability nor by the conditions under which they were learned; (ii) the neural responses showed a larger N100 for hard “words” in the later phase of exposition (second block) of the implicit (vs. explicit) condition, while it was reduced in the second block (vs. the first block) for the easy “words” in the implicit condition; (iii) easy “words” elicited larger N400-like amplitudes than hard “words;” and (iv) “words” that have been previously taught (explicit condition) elicited a general reduced N250 relatively to “words” that were completely unknown to the participants (implicit condition).

These findings provide supportive evidence in favor of our hypotheses. Indeed, although the 2-AFC task was not sensitive either to the type of nonsense word or to the learning condition in which they were presented (implicit vs. explicit), the ERP results indicated that both TPs and instructions affect the electrophysiological correlates of SL in different latency windows. The absence of statistically significant differences across experimental conditions in the 2-AFC tasks indicate that the prior knowledge of the to-be-learned regularities or about the structure of the auditory stream do not suffice to promote a boost in SL performance, as observed in previous studies (e.g., Howard and Ballas, 1980; Reber et al., 1980; Song et al., 2007; Turk-Browne et al., 2009, 2010; Franco et al., 2011; Bertels et al., 2012, 2015; Sanchez and Reber, 2013; Batterink et al., 2015a,b). This might be closely tied to the strength of contingencies of the SL task used in the current article such as the use of a higher number of “words” (eight) when compared to previous SL studies (four in Cunillera et al., 2009; Batterink and Paller, 2017, or Saffran et al., 1996a, and six in Abla et al., 2008; Batterink et al., 2015a,b, or Sanders et al., 2009), that were repeated fewer times (60 repetitions-30 per block) as compared to the abovementioned SL studies (i.e., over 100 repetitions of each “word” in most of the cases). The use of “words” with different TPs might also have contributed to making behavioral effects across the distinct learning conditions harder to find. Indeed, as mentioned in the Introduction, lower TPs introduce a higher level of uncertainty in the input as the same syllable can occur in different “words” in different positions, which could have made the triplets more difficult to extract. Moreover, it is also important to note that the presentation of “words” with TPs of 1.00 and TPs of 0.50 in the same auditory stream might also have contributed to making SL more challenging in general as it introduces important changes in the distributional properties of the input. Note that, besides the different TPs, the fact that a given syllable might occur, or not, in different “words,” as in the case of the hard and easy “words,” also creates a greater variability in the stream, as compared to that created when all the words present TPs of 1.00. If we consider that SL also occurs through the integration of information across exemplars, the higher the diversity of these exemplars in the stream, the more difficult the integration will be, which might also have hampered SL in our procedure (see Thiessen et al., 2013; and also Hasson, 2017). Nevertheless, the low sensitivity of the 2-AFC tasks to capture the effects of words’ predictability and task instructions converge with an increasing number of studies showing that this recognition task might not be well-suited to assess SL. Indeed, since it asks participants to make explicit judgments about regularities that are expected to be acquired implicitly (i.e., without intention and awareness), this might create not only a mismatch between the “mode of learning” and the “mode of assessing” this knowledge but it also leaves room for other meta-cognitive or strategic factors to affect the results (for a discussion see Siegelman et al., 2017a).

The use of the ERP methodology has proven to be a particularly useful tool to cope with these limitations and also to shed light on the issues under investigation. Specifically, the neural results obtained throughout the familiarization phase showed that the type of nonsense word, the type of instructions, and the amount of exposure to the auditory stream modulated specific ERP components. Previous studies suggested the N100 as a possible “marker” of online segmentation (e.g., Sanders and Neville, 2003; Sanders et al., 2002; Abla et al., 2008), which is complemented with the observation of an enhancement of the N100 amplitude in the first element of a successfully segmented triplet (triplet onset effect). However, no effects of type of “word” or task instructions were found in our results, but a significant effect emerged from the triple interaction between the type of “word,” instructions, and exposure time. The sensitivity of the N100 component to various factors present in SL tasks could be the reason why the literature has yielded divergent results (e.g., De Diego Balaguer et al., 2007; Buiatti et al., 2009; Cunillera et al., 2009). On the one hand, the N100 amplitude reductions have been found in tasks with short stimulus onset asynchrony (e.g., Pereira et al., 2014) or in continuous speech presentations compared with non-predicted words (e.g., Astheimer and Sanders, 2011), whereas other studies have failed to observe the N100 effects, showing a moderated learning performance and a reliable N400 effect, but no modulations of the N100 (e.g., Cunillera et al., 2006). This suggests that the N100 might be sensitive to certain features of the task or stimuli. On the other hand, SL studies that found significant N100 modulations also considered the effect of other variables, such as individuals’ learning level (accuracy scores) or the amount of exposure to the stimuli (i.e., initial vs. final task blocks), and they found increased N100 amplitude in “expert learners” during the first part of the learning phase, while “middle learners” only showed an enhanced N100 amplitude in the last part of the task (e.g., Abla et al., 2008). Interpreting our results in the light of Abla et al.’s (2008) discussion, it is possible to hypothesize that the cross-over effect of learning condition, type of “word,” and block (but not each main factor separately) would be explained by a transient learning effect, by which the N100 effects would evolve during the familiarization phase. The N100 attenuation might only be emerging for easy “words” in the implicit task, since the sequences with higher TPs may be more easily segmented when there is no* a priori* knowledge of the stimuli or the structure of the stream. As for the enhancement found over the second block for the hard “words” in the implicit condition, this could also be interpreted as showing that lower TP “words” would need more repetitions to-be-learned, so that they would start evoking larger amplitudes later in time under implicit conditions. However, this difference would not arise in the explicit condition, in which the prior instructions would tend to limit the differential impact of practice and level of difficulty. These results match our hypothesis of the N100 is a learning index whose effects evolve during the familiarization phase and support previous claims of temporal N100 effects as marking the discovery of the structure in a continuous stream (see Abla et al., 2008).

In the N400 ERP component, we found, however, an effect of type of “word” showing that easy “words” elicited larger amplitudes as compared to hard “words” (see Figure 4), which might suggest facilitated access to these specific words’ representations in memory and/or more successful integration of those representations in higher-order language structures (for a review see Lau et al., 2008). The present study is one of the first reporting electrophysiological evidence that word-like units characterized by higher TPs become more easily extracted than units with lower TPs in the context of a continuous, structured speech stream. Also, it corroborates the idea that electrophysiological responses can reflect the brain’s ability to compute TPs and that they can be reliably used to track the online assimilation of statistical regularities embedded in the input. Since the N400 is sensitive to the frequency of occurrence of units and their degree of contextual predictability (e.g., Lau et al., 2008), this finding also shows our capacity to disentangle highly predictable from low predictable sequences, even in the absence of other cues (e.g., stressed syllables, pauses). However, this electrophysiological effect of the type of nonsense word contrasts with the absence of significant differences in the behavioral indices of recognition of easy vs. hard “words” in the explicit assessment of SL. On one hand, this evidence indicates that behavioral measures of SL, as obtained from the 2-AFC task, might not inform on the nature and extent of the cognitive processes underlying SL. Even though our results showed that the average performance level of recognition is close to the score of the low learners in Abla et al. (2008; 58.62%), our results did show significant N400 differences. On the other hand, this shows that the capacity to decode word-like units from a continuous auditory input, and the capacity to explicitly retrieve these units from long-term memory, may represent two interrelated but distinct processes. The divergent results between the indirect (i.e., online ERP responses) and direct (i.e., offline, behavioral responses) measures of SL might also indicate that the two measures might tap into different neurocognitive processes, and highlight the advantage of combining both approaches in SL research.

The fact that the N400 effect was independent of the learning conditions did not support our hypotheses. This result seems to suggest that the emergence of a pre-lexical trace of words’ representation in the brain based on the extraction of TPs is reached regardless of conscious processes (i.e., the fact that participants have or not prior knowledge about the to-be-learned regularities). Furthermore, we did not observe a significant effect of the block (or any interaction with it), which contradicts the results of Abla et al. (2008). As discussed above, a short exposure time would be a limitation of our design, so a possible block effect could be hindered by a short number of repetitions during the familiarization phase, leading to weak statistical effects. Nonetheless, and together with the N100 results, we could consider an alternative explanation based on the assumption that implicit and explicit instructions lead to word segmentation but relying on different mechanisms (see Daltrozzo and Conway, 2014). Under that hypothesis, larger N400 amplitudes would index the result of the word segmentation process (by tracking the implicit statistical probabilities embedded in the input stream), but without being sensitive to these implicit/explicit mechanisms in which the process is based on. Again, the conclusion of whether N400 effects are sensitive to the learning mechanisms and their temporal courses is precluded by the short familiarization phases.

In the 200–300 ms latency range, we found a reduced N250 in the explicit vs. implicit learning condition (see Figure 4). A negative deflection in this latency range has been occasionally reported in the SL literature (e.g., Mandikal-Vasuki et al., 2017b), although with divergent functional interpretations. That is probably because these negative waves might encompass overlapped subcomponents, present or absent depending on task demands (Näätänen and Picton, 1986). As far as the SL tasks used in this article involved the exposition to auditory streams made of the repetition of nonsense words, the results found here cannot be directly compared to those of other studies revealing earlier N2 components related with word-context relations (e.g., van den Brink et al., 2001). It is not plausible that the N250 effect found here is related to deviant stimuli processing (mimicking the traditional MMN effects; see Koelsch et al., 2016), nor linked to the tracking of TPs since the effect only emerges from the comparison of implicit vs. explicit conditions. However, variations of the N250 amplitude have been found as a function of sound complexity and familiarity (e.g., Čeponiené et al., 2001; Vidal et al., 2005). For example, Čeponiené et al. (2001) found a larger N250 for complex sounds than for pure tones. Hence, it is reasonable to anticipate that explicit instructions about the to-be-learned “words” make syllables’ sequences of the auditory stream more familiar, and, thus, less complex as compared to the sequences presented without any further information (implicit condition). Besides, the auditory stream presented during the exposure phases of each of the SL tasks used in our work implies the repetitive presentation of stimuli. This type of presentation elicits a negative wave around 250 ms after stimulus onset, the “basic” N2 ERP (Näätänen and Picton, 1986), a component sensitive to attention deployment, as is reported in the early N2 in Bertoli and Probst (2005). Indeed, in their study, repetitive syllable’ presentations during the detection task-evoked a larger N250 reflecting stimuli inhibition, whilst a greater attention deployment would imply a reduction of the component. That is also the case for the N2 effect reported in Kóbor et al. (2018), in which an N2 reduction related to sequential and SL was found. Interestingly, and of special interest to this discussion regarding SL, the authors found that N2 effects were not necessarily dependent on explicit knowledge. Thus, in the context of our experiment, it is not possible to directly explain the effect on the N250 amplitude by the mere instruction factor. However, possibly the transition from the implicit to the explicit SL task made individuals aware that “word” information was relevant for the task even though that information was not provided, causing the syllable stream to recruit more attention resources in the explicit than in the implicit condition, thereby accounting for the N250 reduction in the explicit SL task. Challenging this interpretation, Mandikal-Vasuki et al. (2017b), who investigated auditory SL abilities in children, found that the N250 was increased in musicians relative to non-musician children. They considered that one possible interpretation is that the N250 is affected by the deployment of attention in the first tone, indicating a correct word segmentation. They proposed that the N250 reflects a prediction process involving higher-order recruitment of attentional resources. The present results can provide an additional interpretation if considered together with the results of Mandikal-Vasuki et al. (2017b): the prediction of the third tone (vs. the first tone) and explicit instructions (vs. implicit condition) would result in a reduced N250, possibly caused by the recruitment of attentional resources. Explicit instructions and awareness that a recognition test will be surely presented at the end of the explicit familiarization task, as it was presented in the previous implicit condition, might have made this stream more relevant for the participants and may have led to the allocation of greater attentional resources and, consequently, to a reduction in the N250 amplitude.

## Conclusion

We investigated the relationship between the cognitive mechanisms underpinning SL and the higher-order evaluative processes characterizing the overt responses in post-learning tasks. We also intended to clarify the role of explicit instructions, the relative difficulty of the “words” as defined by the TPs between their component syllables, and the impact of the practice on the neural correlates of SL. Limitations of the 2-AFC tasks to assess learning suggest that other measures should be used to complement behavioral assessments, like the Rapid Serial Visual Presentation task (RSVP; Kim et al., 2009), that allows a reliable distinction of participants as a function of behavioral performance.

We provided evidence that ERPs can be effective online indices of word segmentation that bring to light some effects of the learning conditions that otherwise would be impossible to grasp. Specifically, modulations of the N400 indicate more efficient segmentation for “words” with higher TPs, whilst effects on the N250 component indicate that instructions lead to deploy more attentional resources. Together, the results suggest, on the one hand, that explicit instructions have no direct effects in improving learning in those conditions in which the extraction of statistical regularities is more difficult, as during the processing of “words” with lower TPs and, on the other hand, that awareness is not crucial for the process of word extraction to occur. Despite the existence of conflicting findings in the N100 literature, we found moderate evidence suggesting that the transient effect in the N100 is an indicator of the moment in which learning occurs. Future research may benefit from this evidence to better understand which experimental conditions facilitate SL (for example, “words” with high TPs as compared to “words” with low TPs), as well as if it might be sensitive to individual differences (e.g., high vs. low learners). Future experimental designs should also include longer exposure times to elucidate if the N100 is indeed a reliable learning index and how N400 amplitude modulations reflect the time course of SL.

## Data Availability Statement

Behavioral and ERP data are available at Figshare https://doi.org/10.6084/m9.figshare.12789356.v1. All the stimuli materials are available on request to the corresponding author.

## Ethics Statement

The studies involving human participants were reviewed and approved by Ethics Committee of University of Minho, Braga, Portugal (SECSH 028/2018). The patients/participants provided their written informed consent to participate in this study.

## Author Contributions

AS and LJ conceived and designed the study. F-JG-D and HO implemented the experiment and collected the data. F-JG-D and MV analyzed the data. AS wrote the manuscript. All authors contributed to the article and approved the submitted version.

## Conflict of Interest

The authors declare that the research was conducted in the absence of any commercial or financial relationships that could be construed as a potential conflict of interest.
